# Delayed Administration of N-Acetylcysteine Blunts Recovery After an Acetaminophen Overdose Unlike 4-Methylpyrazole

**DOI:** 10.1007/s00204-021-03142-9

**Published:** 2021-08-22

**Authors:** Jephte Y. Akakpo, Matthew W. Jaeschke, Anup Ramachandran, Steven C. Curry, Barry H. Rumack, Hartmut Jaeschke

**Affiliations:** 1 Department of Pharmacology, Toxicology & Therapeutics, University of Kansas Medical Center, Kansas City, Kansas, USA; 2 Division of Clinical Data Analytics and Decision Support, and Division of Medical Toxicology and Precision Medicine, Department of Medicine, University of Arizona College of Medicine - Phoenix, Phoenix, AZ, USA; 3 Department of Emergency Medicine and Pediatrics, University of Colorado School of Medicine, Aurora, CO, USA

**Keywords:** acetaminophen, drug hepatotoxicity, regeneration, mitochondrial biogenesis, Fomepizole, N-acetylcysteine

## Abstract

N-acetylcysteine (NAC) is the only clinically approved antidote against acetaminophen (APAP) hepatotoxicity. Despite its efficacy in patients treated early after APAP overdose, NAC has been implicated in impairing liver recovery in mice. More recently, 4-methylpyrazole (4MP, Fomepizole) emerged as a potential antidote in the mouse APAP hepatotoxicity model. The objective of this manuscript was to verify the detrimental effect of NAC and its potential mechanism and assess whether 4MP has the same liability. C57BL/6J mice were treated with 300 mg/kg APAP; 9h after APAP and every 12h after that, the animals received either 100 mg/kg NAC or 184.5 mg/kg 4MP. At 24 or 48h after APAP, parameters of liver injury, mitochondrial biogenesis and cell proliferation were evaluated. Delayed NAC treatment had no effect on APAP-induced liver injury at 24h but reduced the decline of plasma ALT activities and prevented the shrinkage of the areas of necrosis at 48h. This effect correlated with down-regulation of key activators of mitochondrial biogenesis (AMPK, PGC-1α, Nrf1/2, TFAM) and reduced expression of Tom 20 (mitochondrial mass) and PCNA (cell proliferation). In contrast, 4MP attenuated liver injury at 24h and promoted recovery at 48h, which correlated with enhanced mitochondrial biogenesis and hepatocyte proliferation. In human hepatocytes, 4MP demonstrated higher efficacy in preventing cell death compared to NAC when treated at 18h after APAP. Thus, due to the wider treatment window and lack of detrimental effects on recovery, it appears that at least in preclinical models, 4MP is superior to NAC as an antidote against APAP overdose.

## INTRODUCTION

Acetaminophen (APAP) is an analgesic and antipyretic drug that is an active ingredient in many over the counter and prescription medications and is generally considered safe at therapeutic doses (Jaeschke, 2015). However, toxicity can be caused by multiple supratherapeutic doses or an acute, high overdose. APAP overdose is responsible for 56,000 emergency room visits resulting in 500 deaths every year (Bernal et al., 2010). Thus, APAP overdose is the leading individual cause of acute liver failure (ALF) in the United States for many years (Stravitz and Lee, 2019). N-acetylcysteine (NAC) is the only FDA-approved antidote currently available against APAP-induced hepatotoxicity (Rumack and Bateman, 2012); NAC is very effective in humans when administered within 8 to 10 h after APAP ingestion, i.e., before the onset of severe liver injury (Rumack et al., 1981; Smilkstein et al., 1988). This is due to the fact that NAC protects by replenishing hepatic glutathione stores which are depleted by its scavenging of the reactive APAP metabolite, N-acetyl-p-benzoquinone imine (NAPQI) (Corcoran et al., 1985; Corcoran and Wong, 1986). These events occur early after an overdose. Unfortunately, many APAP overdose patients present late to the emergency department, i.e., when most of the drug has been metabolized and the injury phase is initiated. At these later time points, any GSH synthesized through NAC administration will mainly act as a scavenger for peroxynitrite inside mitochondria (Cover et al., 2005; Saito et al., 2010b), and its efficacy will decline the longer NAC treatment is delayed (Smilkstein et al., 1988). In fact, late NAC treatment hypothetically could impair regeneration, which would potentially contribute to ALF (Athuraliya and Jones, 2009). The patient may then require a liver transplant to survive (Stravitz and Lee, 2019). Despite this understanding of its mechanisms of action, NAC is typically given to APAP overdose patients, even when they present more than 10h after APAP ingestion (Fisher and Curry, 2019), because it is the only clinically approved treatment for APAP overdose currently available and is generally considered benign. Furthermore, a randomized controlled trial of NAC in patients presenting late with APAP-induced liver failure reported improved outcomes with NAC therapy (Keays et al. 1991). Treatment with NAC is then continued as per either of two FDA approved protocols: an oral regimen over 72h, or an intravenous regimen over 21h typically until liver enzyme levels decline, which may indicate liver recovery. Most physicians continue NAC in patients with established hepatotoxicity until liver function studies are clearly improving and hepatic encephalopathy, if ever present, has resolved. However, the fact that prolonged administration of NAC in animals can potentially limit hepatocyte regeneration, even when initiated early after an APAP overdose (Yang et al., 2009), necessitates the development of better treatment protocols, personalized to each patient, depending on the presumed APAP dose consumed, especially in cases of severe overdose. In order to do this, understanding of the mechanisms by which delayed NAC modulates liver recovery is required. Therefore, this study focuses on deciphering the mechanisms responsible for the potential detrimental effects of persistent delayed NAC treatment after APAP overdose in the standard animal model. Our previous reports showed that 4-methylpyrazole (4MP), an antidote against methanol and ethylene glycol poisoning (McMartin et al., 2016), is highly effective as an antidote against APAP overdose in mice (Akakpo et al., 2018, 2019, 2020) and in preventing oxidative APAP metabolism in human volunteers (Kang et al., 2020). Thus, this study also investigates the continuous delayed treatment of 4MP after an APAP overdose, as an alternate approach to prevent development of ALF.

## MATERIALS AND METHODS

### Animals and experimental design.

All animal experiments were approved by the University of Kansas Medical Center Institutional Animal Care and Use Committee and conducted in compliance with the National Research Council for the care and use of laboratory animals. Eight- to 12-week-old male C57BL/6J mice were purchased from Jackson Laboratories (Bar Harbor, ME) and housed in the KUMC vivarium maintained at a constant temperature of 20–22°C and kept on a 12h light and dark cycle. Prior to beginning treatment, mice were fasted overnight and then administered an intraperitoneal dose of 300 mg/kg APAP (Sigma-Aldrich, St. Louis, MO), dissolved in warm saline. NAC (Sigma-Aldrich) or 4MP (Sigma-Aldrich) were also dissolved in saline and administered IP as an initial bolus dose (100 mg/kg NAC; 184.5 mg/kg 4MP) 9h after APAP, followed by a maintenance dose (100 mg/kg or 123 mg/kg, respectively) every 12h until euthanasia. The 4MP doses for mice represent human equivalent doses (HED) of 15 and 10 mg/kg, which are clinically approved doses for methanol poisoning. Following all treatments, mice were euthanized under isoflurane anesthesia at 24 or 48h after APAP. Blood was drawn into a heparinized syringe, livers were removed, then the tissues were either immediately flash frozen in liquid nitrogen and subsequently stored at −80°C or fixed in formalin for liver histology.

### Biochemical analyses.

After centrifugation of heparinized blood samples at 16,000× g for 5 min at 4°C, plasma ALT levels were measured using an ALT assay kit (Pointe Scientific, MI), as per the manufacturer’s instructions. The assay for GSH was performed using a modified Tietze method, as described in detail (Jaeschke and Mitchell, 1990).

### Histology and TUNEL staining.

Following formalin fixation, livers were processed, and paraffin-embedded blocks sectioned at 5 μm thickness and were placed on slides. Sections were stained with hematoxylin and eosin (H&E) to evaluate necrosis. The percent of necrosis was quantified by comparing areas of necrosis to that of the entire section. DNA fragmentation was assessed in liver sections by the terminal deoxynucleotidyl transferase-mediated dUTP nick end-labeling (TUNEL) assay carried out using the In-Situ Cell Death Detection Kit, AP (Roche Diagnostics, Indianapolis, IN).

### Immunostaining of liver sections.

Immunostaining was performed on paraffin embedded liver tissue, sectioned at 5 μm thickness. Tissue was first deparaffinized, dehydrated, and then blocked with 5% normal goat serum. This was followed by overnight incubation with 1:200 diluted primary rabbit antibodies that include anti-PCNA (Cat. # sc-7907, Santa Cruz, Dallas, TX), anti-Tom 20 (Cat. # sc11415, Santa Cruz, Dallas, TX), anti-P-AMPK (Cat. #2531; Cell Signaling, Boston, MA), anti-TFAM (Cat. # ab-252432, Abcam, Cambridge, MA) or anti-PGC-1α antibody (Cat. # ab-722330, Abcam, Cambridge, MA). The next day, sections were washed in PBS, followed by application of the secondary antibody, Alexa Fluor 594-conjugated goat anti-rabbit antibody (Cat. # A11037, Life Technologies, Eugene, OR). After staining nuclei with DAPI containing mounting medium (Life Technologies), slides were imaged with a Zeiss Axiovert inverted fluorescence microscope (Carl Zeiss AG, Jena, Germany). Quantitation of the fluorescence intensity signals was done by measuring the mean grey values of the signal from each tissue section with ImageJ(http://rsb.info.nih.gov/ij/).

### Western blot analysis.

Liver tissues were homogenized in tissue lysis buffer (20 mM HEPES, pH 7.0; 2 mM EGTA, 1 mM EDTA, 1% Triton X-100, 10% glycerol, 150 mM NaCl, and 20 mM glycerol-2-phosphate) and centrifuged at 20,000xg at 4 °C for 5 min to remove the cell debris and isolate proteins. The concentration of protein was determined using the BCA protein assay as described (Bajt et al. 2000). Then, proteins were denatured at 95°C for 5 min before separation by electrophoresis on 10–12% sodium dodecyl sulfate (SDS)-polyacrylamide gel and transferred to polyvinylidene fluoride (PVDF) membranes at room temperature for 1h. The PVDF membranes were then incubated overnight in the cold room with 1:1000 diluted primary rabbit antibodies, including anti-P-AMPK (Cat. #2531; Cell Signaling), anti-AMPK (Cat. # #2532; Cell Signaling), anti-rabbit NRF1(Cat. # 2772; Cell Signaling Technology, Danvers, MA), anti-NRF2 (Cat # 5318; Cell Signaling Technology) and anti-Keap1 (Cat # 612245; BD Biosciences, San Diego, CA). A horseradish peroxidase-coupled anti-rabbit IgG (Cat. # sc2030; Santa Cruz, Dallas, TX) was used as secondary antibody (1:5000 dilutions), and proteins were visualized by enhanced chemiluminescence on a Licor Odyssey imager (Licor Biosciences).

### Primary human hepatocyte isolation and treatments.

Primary human hepatocytes (PHH) from de-identified liver donors were isolated as described in detail (Xie et al., 2014). PHH were then seeded on collagen coated plates and allowed to attach in a humidified 5% CO_2_ incubator (at 37°C) for 3 hours. Then, PHH were treated with 10 mM APAP and 18h later with 10 mM or 20 mM 4MP or NAC for 48h. All chemicals were dissolved in William’s medium E supplemented with dexamethasone, insulin, and penicillin.

#### Statistical analysis.

Statistical analysis between two groups was performed with the Student’s two-tailed t-test. Statistical analysis between multiple groups was assessed by one-way analysis of variance (ANOVA), followed by Student-Newman-Keul’s test. If the data were not normally distributed, the Kruskal-Wallis test (non-parametric ANOVA) followed by Dunn’s Multiple Comparison Test was used. Differences with values of P< 0.05 values were considered statistically significant. All statistical analyses were performed using SPSS Statistics 25 (IBM Co., Armonk, NY).

## RESULTS

### Persistent late treatment with NAC delays liver recovery after APAP overdose.

**Figure 1:**
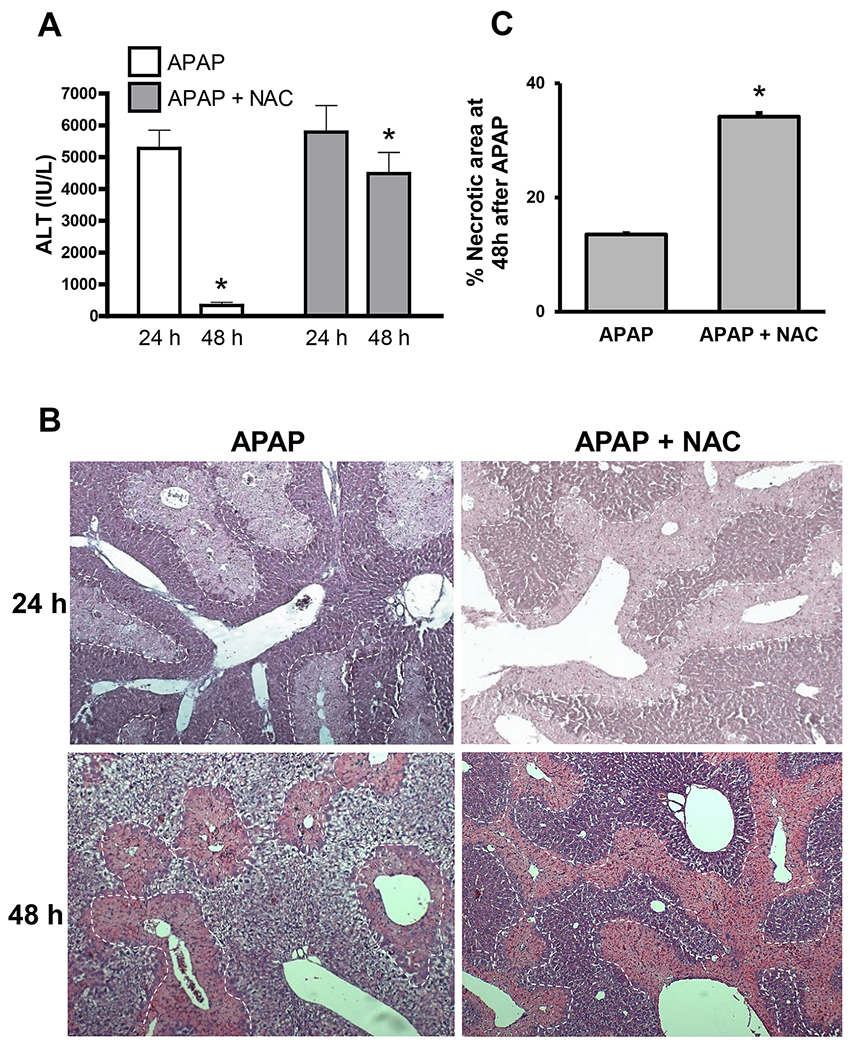
Effect of delayed N-acetylcysteine treatment on liver injury. Mice were treated with 300 mg/kg APAP or saline (10 ml/kg) IP. Then, 9h later, mice received 100 mg/kg NAC IP followed by additional doses every 12h. Blood and liver tissue samples were collected at 24 and 48h post-APAP. (A) Plasma alanine aminotransferase (ALT) activities. (B) H&E-stained liver sections. (C) Area of necrosis at 48 h (%). **Bars represent means ± SE for n = 9 mice per group and time point.** *P<0.05 vs. APAP.

**Figure 2:**
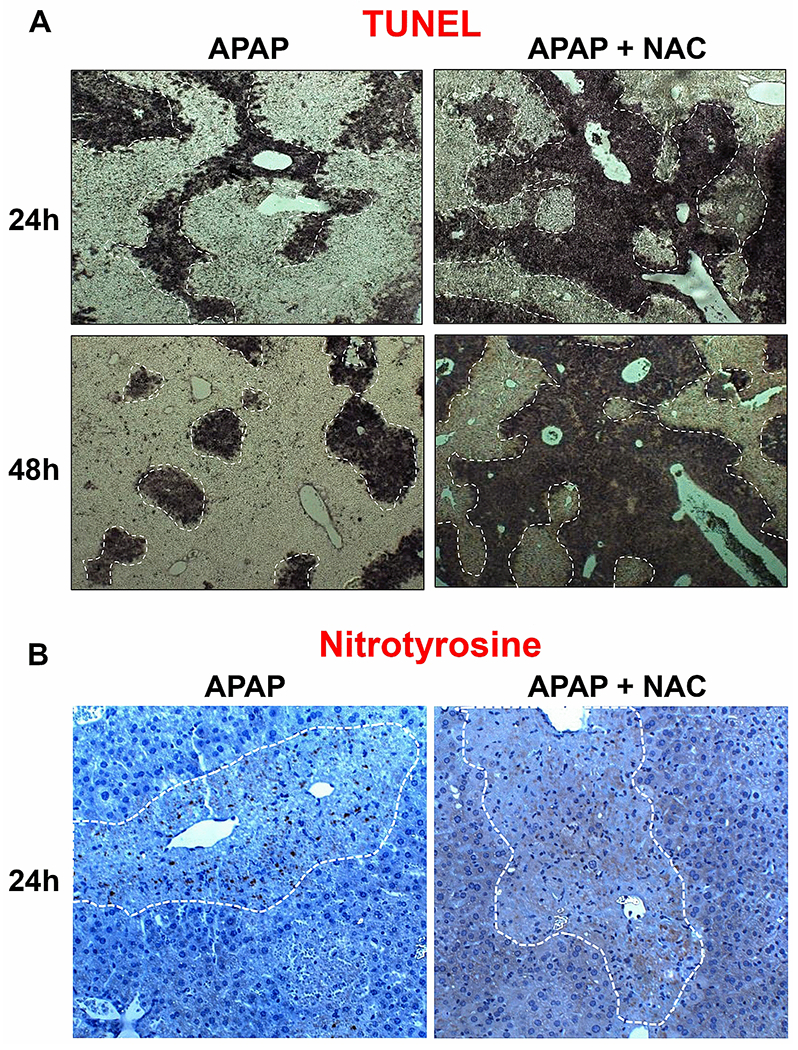
Effect of delayed N-acetylcysteine treatment on DNA fragmentation and peroxynitrite formation. Mice were treated with 300 mg/kg APAP or saline (10 ml/kg) IP. Then, 9h later, mice received 100 mg/kg NAC IP followed by additional doses every 12h. (A) TUNEL-stained liver sections (24, 48h). (B) Nitrotyrosine-stained liver sections (24h).

To evaluate the effect of prolonged late NAC exposure, fasted male C57BL/6J mice were treated with a moderate 300 mg/kg APAP overdose followed by NAC (100 mg/kg; i.p.) 9h later. This time point was selected to not interfere with APAP metabolism or the injury phase and to study the effect of NAC on recovery independent of the injury. After the initial dose, mice were then administered NAC (100 mg/kg) IP every 12h as a maintenance dose until euthanasia. APAP overdose caused significant liver injury by 24h as shown by elevated plasma ALT activities, which decreased close to basal levels at 48h, indicating liver recovery (Figure 1A). In contrast, mice subjected to prolonged late NAC treatment after APAP maintained significant elevated ALT levels at 48h though the levels at 24h were similar to APAP controls (Figure 1A). This different response was also reflected in H&E-stained liver sections, which revealed extensive centrilobular necrosis at the 24h time point with decreasing areas of necrosis by 48h in mice treated with APAP alone (Figure 1B). However, APAP+NAC treated mice maintained the areas of necrosis at 48h (Figure 1B), which were significantly larger than in animals with APAP alone (Figure 1C). In contrast to the clear delay in recovery in the NAC-treated group at 48h, the NAC-treated animals showed close to complete tissue repair similar to the APAP control group at 96h (data not shown). The changes in ALT and liver histology (Figure 1) were also supported by measurement of DNA fragmentation, which is a characteristic feature of APAP-induced necrosis (Gujral et al., 2002). Analysis by TUNEL staining showed significant DNA fragmentation in necrotic areas at 24h after APAP, which partially resolved by the 48h time point (Figure 2A). Again, mice treated with NAC after APAP showed persistence of TUNEL staining at 48h indicating a compromised ability for liver recovery. While nitrosative stress is a feature early during APAP-induced liver injury (Knight et al., 2001, 2002), this is relatively resolved by 48h after APAP, where nitrotyrosine protein adducts are restricted to fragments within the necrotic area (Figure 2B), which presumably came from cells which succumbed to injury earlier. In contrast, however, animals treated with delayed NAC show significant staining for nitrotyrosine protein adducts within hepatocytes around areas of necrosis, even at 48h after APAP, further suggesting a delay in initiation of the recovery process typically seen after APAP alone.

### Prolonged late NAC treatment compromises liver regeneration and mitochondrial biogenesis after APAP overdose.

**Figure 3:**
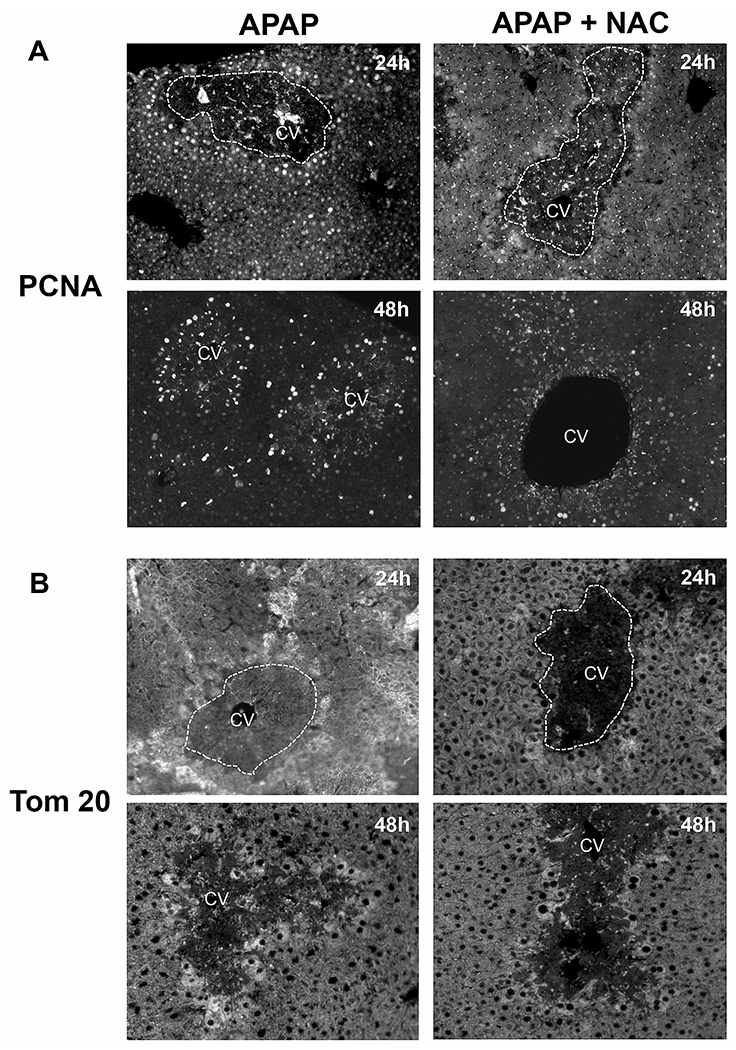
Effect of delayed N-acetylcysteine treatment on regeneration and mitochondrial mass. Mice were treated with 300 mg/kg APAP or saline (10 ml/kg) IP. Then, 9h later, mice received 100 mg/kg NAC IP followed by additional doses every 12h. Representative images of immunofluorescence staining for PCNA (A) and Tom 20 (B) of tissue sections collected at 24 and 48h.

Mitochondrial biogenesis and cell proliferation is critical for survival after APAP-induced liver injury, and this is initiated in surviving hepatocytes around the necrotic area beginning at 24h after a moderate APAP overdose (Du et al., 2017). The effect of delayed prolonged NAC treatment on these molecular events necessary for recovery were then examined by staining liver sections for PCNA, a central component of DNA replication, as well as Tom 20, a component of the mitochondrial protein translocation system and surrogate for mitochondrial mass. In liver sections from mice treated with APAP alone, nuclear PCNA staining was distinctly evident in cells immediately surrounding the area of necrosis by 24h, which was also sustained at the 48h time point (Figure 3A). However, in animals treated with delayed NAC, this intense PCNA staining of peri-necrotic hepatocytes at 24h was largely absent and staining at 48h was also milder compared to APAP alone (Figure 3A). Quantitation of PCNA fluorescence in these liver sections (n=3) showed that compared to the respective APAP-treated animals (100%), the values for APAP+NAC-treated groups were reduced by 62% (P<0.05) at 24h and by 57% (P<0.05) at 48h. Examination of mitochondrial biogenesis using staining for the mitochondrial protein Tom 20 revealed the initiation of mitochondrial biogenesis in cells around the necrotic area by 24h after APAP, which was significantly enhanced by 48h post APAP (Figure 3B). Interestingly, animals treated with delayed NAC had substantially less mitochondrial mass around the area of necrosis at 24h post APAP, and an upregulation of biogenesis only seems to be evident in a few cells by 48h after APAP (Figure 3B). Quantitation of Tom 20 fluorescence in these liver sections (n=3) showed that compared to the respective APAP-treated animals (100%), the values for APAP+NAC-treated groups were reduced by 70% (P<0.05) at 24h and by 54% (P<0.05) at 48h. Overall these data indicate that delayed NAC treatment blunted the induction of mitochondrial biogenesis typically seen in surviving cells around the area of necrosis by 24h after a moderate APAP overdose (300mg/kg) and also compromised liver regeneration.

### Persistent late treatment with NAC significantly impaired AMPK activation.

**Figure 4:**
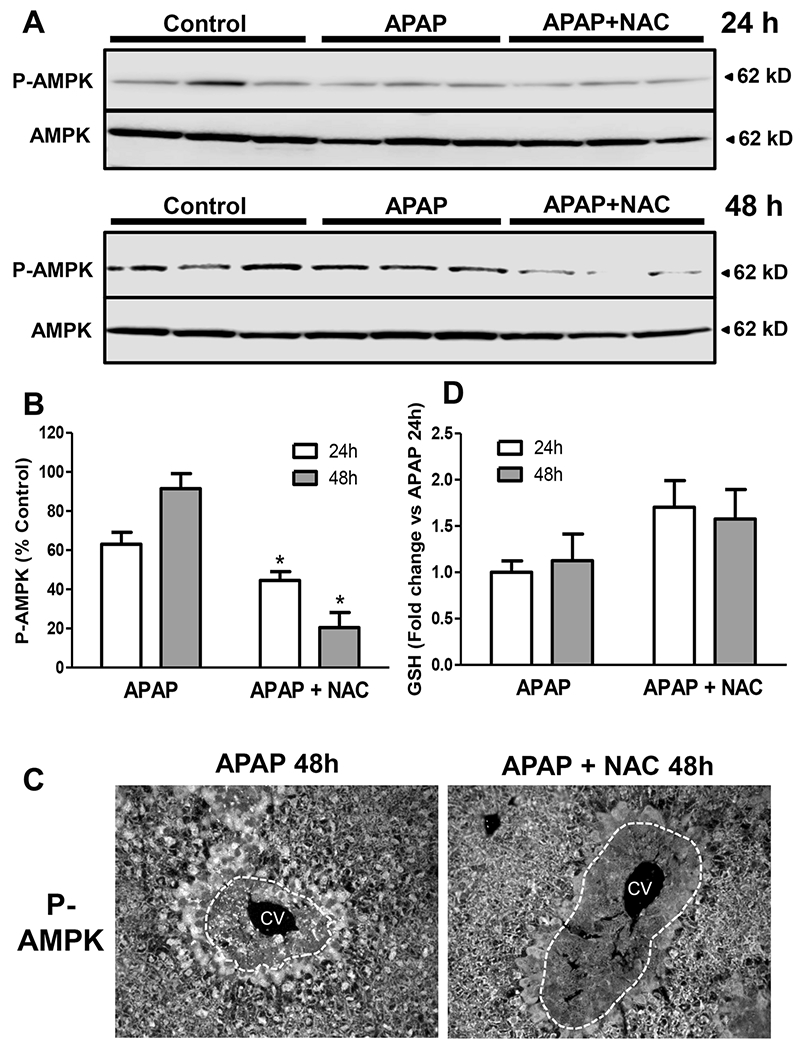
Effect of delayed N-acetylcysteine treatment on AMP-activated protein kinase activation and GSH levels. Mice were treated with 300 mg/kg APAP or saline (10 ml/kg) IP. Then, 9h later, mice received 100 mg/kg NAC IP followed by additional doses every 12h. Liver samples were collected 24 and 48h post-APAP. (A) Western blot analysis for P-AMPK and AMPK of APAP and APAP+NAC-treated animals. (B) Densitometry of P-AMPK and AMPK western blots. (C) Fluorescence staining for P-AMPK at 48h. (D) Hepatic GSH levels at 24 and 48h. Bars represent means ± SE for n = 3 mice per group and time point. *P<0.05 vs APAP.

Early NAC supplementation enhances hepatic and mitochondrial GSH levels and supports mitochondrial energy metabolism (Saito et al., 2010b). The AMP-activated protein kinase (AMPK) is an energy sensor that regulates cellular metabolism (Long and Zierath, 2006) and has been shown to play a role in liver regeneration after acute liver injury induced by carbon tetrachloride (Huang et al., 2018). An examination of AMPK levels by western blotting during the recovery phase after APAP overdose revealed that P-AMPK levels are around 60% of controls at the 24h time point, but these recover close to control levels by 48h after APAP (Figure 4A,B). In contrast, mice treated with delayed NAC after APAP showed significant suppression of P-AMPK levels at both the 24 and 48h time point (Figure 4A,B), indicating that this could be the cause for the delayed recovery in these mice. The western blot data are supported by immunofluorescence staining for P-AMPK which shows robust activation of AMPK in cells surrounding the necrotic area in mice treated with APAP alone, which was significantly blunted in animals with APAP and delayed NAC administration (Figure 4C). Quantitation of P-AMPK fluorescence in these liver sections (n=3) showed that compared to the respective APAP-treated animals (100%), the values for APAP+NAC-treated groups were reduced by 75% (P<0.05) at 24h (data not shown) and by 76% (P<0.05) at 48h. It has been demonstrated that AMPK activation can be influenced by hepatic glutathione levels, with chronic glutathione depletion activating AMPK (Chen et al., 2016). Since NAC supplementation typically enhances hepatic glutathione levels, this late increase in hepatic glutathione could be suppressing P-AMPK. In fact, examination of the hepatic glutathione content indicates that, as expected, NAC-treated animals had increased levels of total GSH when compared to animals treated with APAP alone at both 24 and 48h (Figure 4D) when P-AMPK levels were suppressed.

### Delayed NAC-induced suppression of P-AMPK has wide ranging effects on mitochondrial biogenesis.

**Figure 5:**
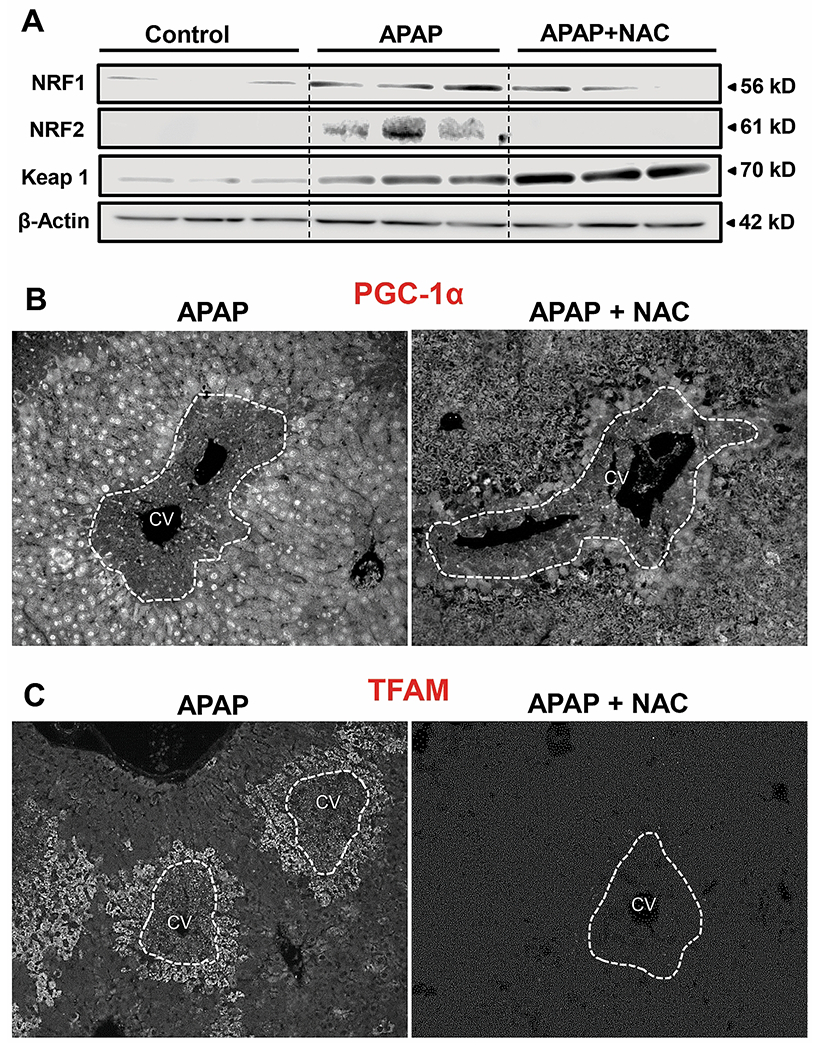
Effect of delayed N-acetylcysteine treatment on Nrf2 activation and mitochondrial biogenesis. Mice were treated with 300 mg/kg APAP or saline (10 ml/kg) IP. Then, 9h later, mice received 100 mg/kg NAC IP followed by additional doses every 12h. Liver samples were collected 48 h post-APAP. (A) Western blot analysis for NRF1, NRF2, Keap1, and β-Actin. Fluorescence staining for PGC-1α (B) and TFAM (C) at 48h after APAP.

The next series of experiments examined consequences of the P-AMPK suppression by delayed NAC and evaluated mitochondrial biogenesis and liver recovery. APAP treated mice showed upregulation of NRF1 (Figure 5A), a mediator of mitochondrial biogenesis (Jornayvaz and Shulman, 2010), by 48h, accompanied by elevation in NRF2, which also plays a role in liver regeneration (Dayoub et al., 2013). In contrast, delayed NAC suppressed elevations in both NRF1 & 2, but enhanced levels of the NRF2 binding partner Keap1, which sequesters NRF2 in the cytosol (Figure 5A). Examination of PGC-1α by immunofluorescence staining showed robust upregulation in cells surrounding areas of necrosis by 48h after APAP treatment, which was significantly blunted by delayed NAC treatment (Figure 5B). Quantitation of PGC-1α fluorescence in these liver sections (n=3) showed that compared to the respective APAP-treated animals (100%), the values for APAP+NAC-treated groups were reduced by 69% (P<0.05) at 48h. Staining for the mitochondrial transcription factor, TFAM, which is essential for mitochondrial DNA maintenance (Larsson et al., 1998), also demonstrated robust upregulation in surviving hepatocytes around areas of necrosis by 48h after APAP, and this was again absent in animals subjected to delayed NAC treatment (Figure 5C). Quantitation of TFAM fluorescence in these liver sections (n=3) showed that compared to the respective APAP-treated animals (100%), the values for APAP+NAC-treated groups were reduced by 99% (P<0.05) at 48h. Thus, suppression of AMPK activation by delayed NAC has wide ranging effects on mitochondrial biogenesis and liver recovery.

### Delayed treatment with 4-methylpyrazole hastens liver recovery after an APAP overdose.

**Figure 6:**
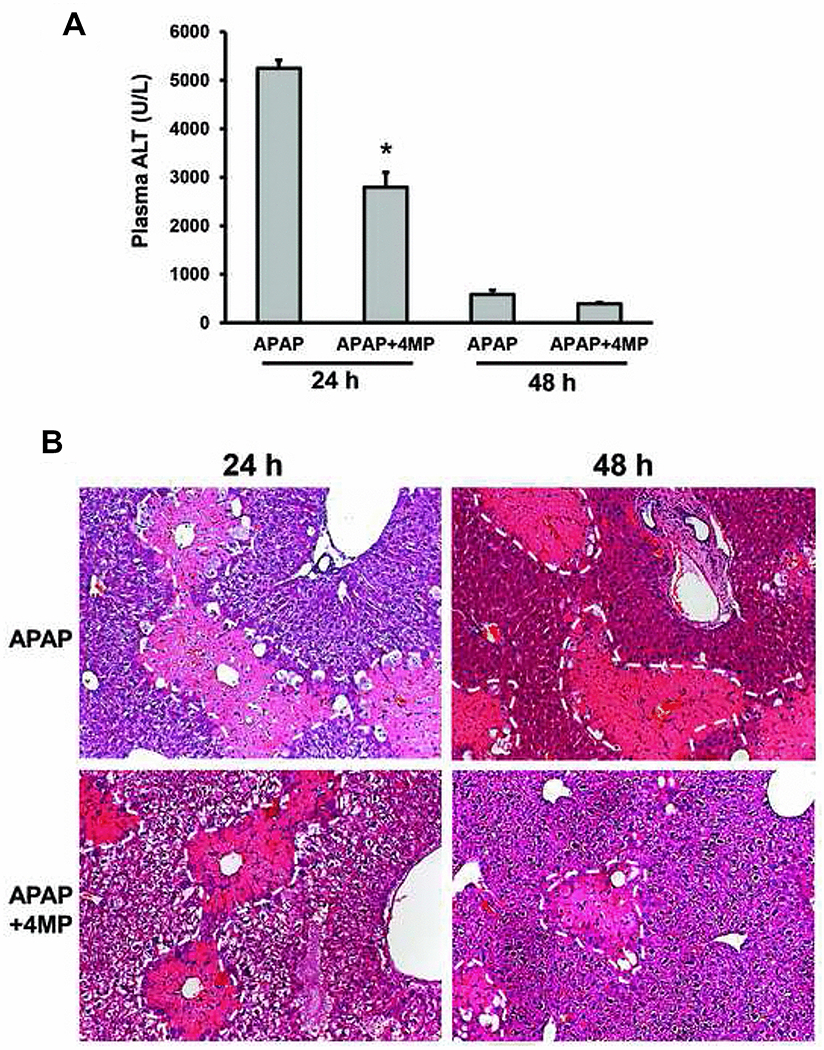
Effect of delayed 4-methylpyrazole treatment on liver injury. Mice were treated with 300 mg/kg APAP or saline (10 ml/kg) IP. Then, 9h later, mice received an initial bolus dose of 184.5 mg/kg 4MP (HED of 15 mg/kg) IP followed by maintenance doses of 123 mg/kg 4MP (HED of 10mg/kg) every 12h. Blood and liver tissue were collected at 48h post-APAP. (A) Plasma alanine aminotransferase (ALT) activities after APAP treatment; (B) H&E staining of liver tissue sections. **Bars represent means ± SE for n = 6 mice per group.** *P<0.05 vs. APAP.

**Figure 7:**
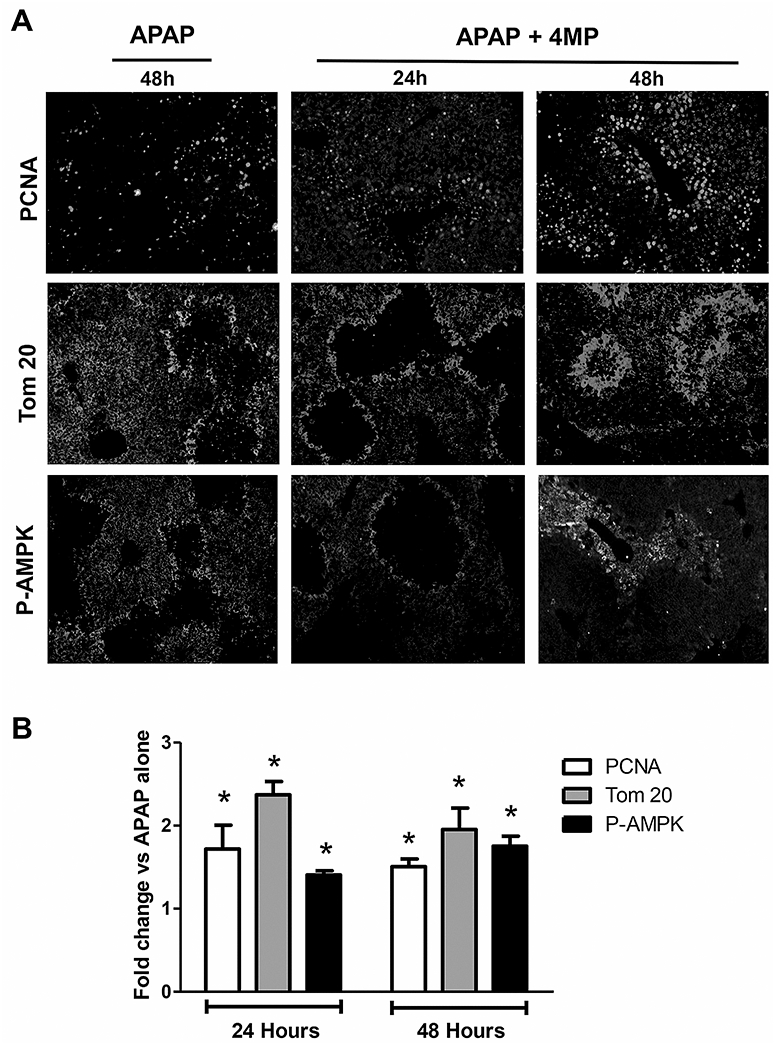
Effect of delayed 4-methylpyrazole treatment on liver recovery. Mice were treated with 300 mg/kg APAP or saline (10 ml/kg) IP. Then, 9h later, mice received an initial bolus dose of 184.5 mg/kg 4MP (HED of 15 mg/kg) IP followed by maintenance doses of 123 mg/kg 4MP (HED of 10mg/kg) every 12h. (A) Representative images of immunofluorescence staining for PCNA, Tom 20 and P-AMPK of liver sections collected at 24 and 48h post-APAP. (B) Quantitation of fluorescence signals. Bars represent means ± SE of 3 animals per group. *P<0.05 vs APAP.

We have earlier demonstrated that the repurposed drug 4MP (Fomepizole) has significant protective effects against APAP-induced hepatotoxicity in mice and inhibits oxidative APAP metabolism in humans (Akakpo et al., 2018, 2019; Kang et al., 2020). Therefore, we evaluated if delayed 4MP treatment had detrimental effects comparable to NAC. Mice were treated with a moderate APAP overdose (300mg/kg) and administered 184.5 mg/kg 4MP (HED of 15 mg/kg) 9h later and maintenance doses of 123 mg/kg (HED of 10 mg/kg) every 12h thereafter, similar to NAC treatment. Mice were then evaluated at 24 and 48h post APAP. Animals treated with delayed 4MP showed significantly lower ALT levels at 24h and a trend to lower values at 48h when compared to APAP alone, indicating faster recovery from liver injury (Figure 6A). This beneficial effect was confirmed by liver histology, which revealed significantly smaller areas of necrosis in 4MP treated mice compared to those with APAP alone (Figure 6B). In addition, when comparing staining for PCNA (DNA replication), Tom 20 (mitochondrial mass), and P-AMPK (energy sensor) in 4MP-treated animals with APAP alone, it is quite obvious that 4MP did not inhibit liver recovery (Figure 7A: 48h APAP and 24/48h APAP+4MP and Figure 3: 24h APAP), as was observed with NAC (Figure 3). In contrast, 4MP enhanced mitochondrial biogenesis and regeneration at 48h (Figure 7A). Quantitation of the images indicated that APAP+4MP induced a 50 – 150% (P<0.05) increase over values for APAP alone (Figure 7B). A caveat of quantitating the fluorescence in each section is that it underestimates the changes in the hepatocytes around the area of necrosis, which is better reflected in the immunofluorescence images.

### Late treatment with 4MP provides better protection than NAC in human hepatocytes after APAP overdose.

**Figure 8:**
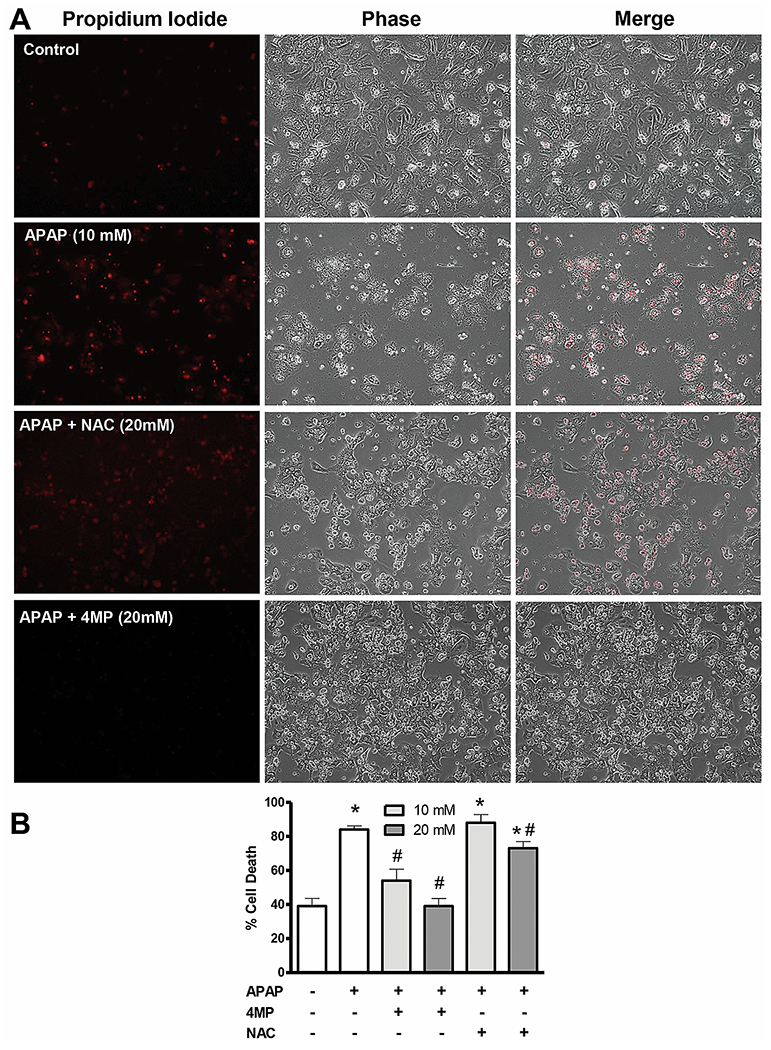
Effect of delayed N-acetylcysteine and 4-methylpyrazole treatment on cell death in human hepatocytes. Freshly isolated primary human hepatocytes were treated with 10 mM APAP. At 18h after APAP, 10 mM or 20 mM 4MP or NAC were added, and cell death was evaluated at 48h. (A) Propidium iodide, phase contrast and merged images of control and treated cells. (B) % of cell death based on ALT release. Bars represent means ± SE for 3 separate cell isolations. *P<0.05 versus Control; ^#^P< 0.05 versus APAP.

To confirm the relevance of the findings in mice to humans, experiments were carried out in primary human hepatocytes exposed to APAP. Isolated cells were treated with 10mM APAP and cell death examined by propidium iodide (PI) staining and % ALT release from cells 48h later. Some cells were also treated with NAC or 4MP (10 or 20mM) 18h after APAP to compare effects on cell death. Treatment with 10mM APAP resulted in significant cell death as seen by both PI staining (Figure 8A) and enhanced ALT release (Figure 8B). While both concentrations of 4MP provided significant protection against APAP-induced cell death (Figure 8A,B), only the highest NAC concentration of 20mM was partially effective (Figure 8B).

## DISCUSSION

The objectives of this study were to replicate the detrimental effects of prolonged NAC treatment in mice, explore parallel effects in human liver cells, assess the potential mechanism of this effect, and evaluate whether 4MP, an antidote against APAP toxicity that came more recently into focus, displays different effects on hepatocyte regeneration. Only male mice were used in these studies because previous investigations showed that the mechanism of APAP-induced liver injury is the same in male and female mice (Du et al., 2014; Duan et al., 2020; Masubuchi et al., 2011). However, due to the more extensive induction of glutamate-cysteine ligase, the rate limiting enzyme of GSH synthesis, the faster recovering hepatic GSH levels in female mice scavenge more peroxynitrite, and thus, female mice are less susceptible (Du et al., 2014; Masubuchi et al., 2011). The sensitivity to an APAP overdose in female mice can be brought to the level of male mice if GSH synthesis is inhibited (Masubuchi et al., 2011) or if generally a higher APAP dose is used (Duan et al., 2020).

### Use of N-acetylcysteine as antidote against APAP toxicity.

APAP overdose in patients can be broadly divided into 2 categories: early- and late-presenting patients. Early-presenting patients show generally elevated levels of APAP, and when the APAP concentrations are above the treatment line in the Rumack-Matthew nomogram between 4 and 24h after a single APAP overdose, the patients will be treated with a standard course of 21h IV NAC or 72h oral NAC regimen (Rumack and Bateman, 2012). In the vast majority of cases, the NAC-mediated increase in hepatic GSH levels will effectively scavenge NAPQI and prevent both significant protein adduct formation (Xie et al., 2015) and liver injury (Rumack et al., 1981; Smilkstein et al., 1988). Because there is no liver injury, there is no regeneration, and the potential detrimental effects of NAC are limited to occasional nausea and vomiting or anaphylactoid reactions (Pakravan et al., 2008). The treatment of the second category, late-presenting patients, is more difficult to manage. In these patients, the injury process has already started, as indicated by elevated liver enzyme levels and protein adduct formation (Xie et al., 2015). Because the levels of APAP and its metabolites are generally lower in late-presenting patients at these late time points, drug metabolism and NAPQI formation may be minimal (Xie et al., 2015) when these patients present for treatment. Thus, the beneficial effect of GSH synthesis induced by NAC is limited to scavenging reactive oxygen and reactive nitrogen species (Saito et al., 2010b), which are critical mediators of cell death. Nevertheless, NAC’s effectiveness decreases the longer treatment is delayed (Rumack et al., 1981; Smilkstein et al., 1988). This clinical observation could also be confirmed with primary human hepatocytes (Xie et al., 2014). However, the problem is that if a standard NAC treatment course is initiated in a delayed fashion due to the late arrival of the patient in the hospital, NAC treatment may last beyond the injury phase. Similarly, in patients with severe overdoses, the standard NAC treatment may not be sufficient to prevent liver injury, and NAC treatment will generally be continued until hepatic function is clearly improving (declining ALT levels and no further increase in bilirubin levels) and there is no evidence of hepatic encephalopathy (Fisher and Curry, 2019). This standard of care, at least in part, is based on a randomized clinical trial by Keays et al. (1991). Because of the long half-life of ALT and the delay in obtaining the result from the clinical laboratory, this may result in NAC treatment well past the injury phase. Our preclinical data in a relevant translational model clearly demonstrated the detrimental effect of the delayed NAC treatment beyond the injury phase. Although highly effective when administered early after the APAP overdose in mice (Corcoran et al., 1985; Saito et al., 2010b), NAC treatment at the end of the injury phase and beyond inhibits mitochondrial biogenesis and regeneration. However, it needs to be emphasized that these effects of NAC cause a substantial delay in the initiation of repair but do not prevent recovery in mice. Given the very similar mechanisms of APAP-induced liver injury and recovery in mice and in humans, with just a more prolonged injury process in humans (Jaeschke et al., 2014; Xie et al., 2014; McGill et al., 2012), these effects of NAC during regeneration may also be applicable to patients. In a clinical scenario, this effect of NAC may have the potential to delay tissue repair and, because the timely onset of regeneration is critical for recovery and survival (Bhushan and Ante, 2019), any delay in regeneration may increase the risk for developing acute liver failure. An observational study of patients in whom NAC treatment was started during the peak of injury could provide evidence for or against this effect in humans, which, if present, could raise awareness among clinicians that NAC treatment should be restricted to the injury phase in order to avoid potential negative effects on the recovery.

### Potential mechanisms of NAC-induced delayed recovery.

Our data showed that delayed and prolonged NAC treatment inhibited PCNA expression, a marker of cell proliferation and Tom 20 expression, a marker of mitochondrial mass, at later time points. Mitochondrial biogenesis, which is critical for recovery after APAP hepatotoxicity (Du et al., 2017), is regulated by the activation of PGC1- α, NRF1/2 and TFAM (Canto and Auwerx, 2010). In addition, the most upstream regulator of this cascade is AMPK, which functions as a central mediator of the cellular response to energetic stress and coordinates multiple features of mitochondrial biology, including mitochondrial biogenesis (Herzig and Shaw, 2018). Metabolic sensors such as AMPK have been shown to directly influence PGC-1α activity (Canto and Auwerx, 2009) and, in fact, AMPK activation triggers a PGC-1α-dependent antioxidant response that limits mitochondrial reactive oxygen production (Rabinovitch et al., 2017). Since redox regulation of mitochondrial biogenesis has also been recognized (Kamble et al., 2013; Piantadosi and Suliman, 2012), this suppression of mitochondrial oxidant stress by late NAC treatment could prevent reactive oxygen species from rising to levels needed for signaling mitochondrial biogenesis. Our data showed that all mediators of mitochondrial biogenesis (PGC1-α, NRF1/2 and TFAM) and the most upstream regulator of these events, AMPK, were upregulated during the recovery phase after an APAP overdose, especially in cells around the area of necrosis. The delayed and prolonged NAC treatment downregulated these mediators and thus, severely delayed mitochondrial biogenesis and cell proliferation in these animals. Our data suggest that downregulation of AMPK activation may be the primary target of the detrimental NAC effect we observed. Chronic GSH depletion was shown to activate AMPK (Chen et al., 2016), which raises the possibility that elevated GSH levels caused by NAC treatment may be responsible for the suppression of P-AMPK and, consequently, all downstream effects that result in reduced mitochondrial biogenesis and delayed recovery. Although more detailed mechanistic studies are needed to verify the role of elevated GSH levels in this process, it may not be feasible to avoid this problem of NAC. Thus, the more promising strategy may be to use a different intervention, which does not have this liability.

### Prolonged 4MP treatment and recovery after APAP overdose.

4MP is known as a potent Cyp2E1 inhibitor (Hazai et al., 2002). Thus, co-treatment of 4MP and APAP protected against APAP toxicity in Cyp2E1-overexpressing HepG2 cells (Dai and Cederbaum, 1995), in mice *in vivo* (Akakpo et al., 2018, 2020) and in primary human hepatocytes (Akakpo et al., 2018). The substantial reduction of oxidative metabolite formation of APAP and the almost complete elimination of APAP protein adducts in livers and in kidneys with early 4MP treatment (Akakpo et al., 2018, 2020), clearly indicates Cyp2E1 inhibition as the main mechanism of protection. These findings could be confirmed in human volunteers where 4MP cotreatment with a mild overdose of APAP also reduced the generation of oxidative metabolites of APAP by 90% (Kang et al., 2020). However, even a delayed treatment with 4MP at a time when the metabolism phase of APAP is over, still effectively protects against APAP hepatotoxicity in mice (Akakpo et al., 2019). Under these conditions, the mechanism of protection involves the inhibition of JNK activation and its translocation to the mitochondria (Akakpo et al., 2019). The activation of JNK in the cytosol (Hanawa et al., 2008) and the binding of P-JNK to Sab on the outer mitochondrial membrane (Win et al., 2011), which triggers further impairment of the electron transport chain (Win et al., 2016) and amplifies the mitochondrial oxidant stress (Saito et al., 2010a), are critical features of the pathophysiology of APAP-induced cell death (Ramachandran and Jaeschke, 2019). Thus, 4MP effectively prevents the amplification of the oxidative and nitrosative stress in mitochondria (Akakpo et al., 2019). This mechanism of action explains the efficacy of 4MP against toxicity in human hepatocytes, even when treated 18h after APAP (Figure 8). It is known that JNK activation (6h) and mitochondrial translocation (15h) are much more delayed in primary human hepatocytes compared to mice (Xie et al., 2014). Thus, the time window for 4MP’s beneficial action is considerably longer in human hepatocytes, allowing 4MP to be still effective at these late time points. Interestingly, these data are consistent with clinical data where NAC is highly effective at early time points, but with therapeutic efficacy fading between 8 - 24 h after APAP (Rumack et al., 1981; Smilkstein et al., 1988). Although 4MP has similar therapeutic targets as NAC (Corcoran et al., 1985; Saito et al., 2010b), the differential efficacy can be explained by direct effects of 4MP on Cyp2E1 preventing NAPQI formation, and on JNK preventing the amplification of the oxidative and nitrosative stress, as compared to NAC promoting GSH formation that scavenges NAPQI (Corcoran et al., 1985) and has to be transported into mitochondria to scavenge peroxynitrite (Saito et al., 2010b).

As we replicated and expanded the original observation by Yang et al. (2009) that prolonged NAC treatment beyond the actual injury phase impairs mitochondrial biogenesis and hepatocellular regeneration, it was important to assess whether 4MP treatment may have the same limitations. However, our data consistently showed that even delayed 4MP treatment starting in the middle of the injury phase and beyond did not impair tissue recovery, including expression of PCNA (indicator of DNA replication), Tom 20 (surrogate for mitochondrial mass and biogenesis), and P-AMPK (regulator of cellular energy metabolism). This suggests that delayed 4MP treatment is not only superior to NAC in terms of protection against APAP-induced liver injury but, in contrast to NAC, does not negatively affect regeneration in mice.

In summary, when mice are treated with NAC during the late injury phase and beyond, P-AMPK, PGC-1α, Nrf1/2 and TFAM are down-regulated, resulting in reduced mitochondrial biogenesis and delayed recovery. In contrast, similar 4MP treatment not only showed an extended effective time window compared to NAC in mice and in human hepatocytes but also displayed no detrimental effect on mitochondrial biogenesis and recovery. Thus, given the more limited side effects of 4MP (Rasamison et al., 2020) compared to NAC (Pakravan et al., 2008), the wider time window of 4MP dosing after APAP, and the lack of detrimental effects on recovery, it appears that at least in preclinical models, 4MP is superior to NAC as an antidote against APAP overdose. These observations provide the rationale for extended clinical testing of 4MP as an adjunct treatment to the standard of care NAC in APAP overdose patients, and maybe even as an alternate to NAC in the future.

## List of Abbreviations

ALF: acute liver failure
ALT: alanine aminotransferase
AMPK: AMP-activated protein kinase
APAP: N-acetyl-p-aminophenol, acetaminophen
CYP: cytochrome P450
GSH: glutathione
H&E: hematoxylin and eosin
HED: human equivalent dose
IP: intraperitoneal
JNK: c-jun N-terminal kinase
Keap 1: Kelch-like ECH-associated protein 1
4-MP: 4-methylpyrazole
MPTP: mitochondrial permeability transition pore
NAC: N-acetylcysteine
NAPQI: N-acetyl-p-benzoquinone imine
NRF1: nuclear respiratory factor 1
NRF2: nuclear factor erythroid 2-related factor 2
PCNA: Proliferating cell nuclear antigen
PGC-1α: peroxisome proliferator-activated receptor gamma coactivator 1-alpha
PI: propidium iodide
PHH: primary human hepatocytes
P-JNK: phospho-JNK
TFAM: Transcription Factor A, Mitochondrial
Tom 20: translocase of outer membrane 20
TUNEL: terminal deoxynucleotidyl transferase dUTP nick end labeling
